# Validation of a Commercial ELISA Kit for Non-Invasive Measurement of Biologically Relevant Changes in Equine Cortisol Concentrations

**DOI:** 10.3390/ani14192831

**Published:** 2024-10-01

**Authors:** Elizabeth R. Share, Sara L. Mastellar, Jessica K. Suagee-Bedore, Maurice L. Eastridge

**Affiliations:** 1 Department of Animal Sciences, The Ohio State University, Columbus, OH 43210, USA; eastridge.1@osu.edu; 2 Agricultural Technical Institute, The Ohio State University, Wooster, OH 44691, USA; mastellar.1@osu.edu; 3 Department of Animal and Poultry Sciences, Virginia Tech University, Blacksburg, VA 24061, USA; jksuagee@vt.edu

**Keywords:** cortisol, ELISA, equine, horse, stress, fecal

## Abstract

**Simple Summary:**

Cortisol is a very common hormone measured when studying the stress response. While plasma cortisol is commonly utilized, concerns regarding stress caused by the sampling procedure and whether that sample represents acute vs. chronic stress have paved the way for measuring fecal cortisol metabolites (FCMs). The measurement of FCMs can be costly, and some reagents used require special permission. Commercial ELISA kits have become popular as a cost-effective method of cortisol measurement, but it is crucial to validate if these kits can detect a biologically significant change in the hormone being measured within the animal being studied. In our work, we conclude that the Arbor Assays^TM^ DetectX^®^ Cortisol ELISA kit (K003-H1, Ann Arbor, MI, USA) is a reliable, economic option for the measurement of biologically relevant changes in cortisol in equine plasma and FCMs. This work helps to provide another potential tool for researchers who want to evaluate horse stress and well-being.

**Abstract:**

The measurement of fecal cortisol/corticosterone metabolites (FCMs) is often used to quantify the stress response. The sampling method is relatively non-invasive, reduces concern for elevation of cortisol from the sampling method, and has been shown to measure cortisol more consistently without the daily diurnal rhythm observed in blood. Commercial ELISA (enzyme-linked immunoassay) kits offer benefits over previously validated immunoassay methods but lack validation. The objective of this study was to evaluate a commercial ELISA kit (Arbor Assays^TM^ DetectX^®^ Cortisol ELISA kit, K003-H1, Ann Arbor, MI, USA) and provide analytical and biologic validation of equine fecal and plasma samples. Horses (4 male, 4 female, mean ± SD: 4 ± 5 yr) were transported for 15 min with limited physical and visual contact via a livestock trailer. Blood and fecal samples were collected pre- and post-transportation. Parallelism, accuracy, and precision tests were used to analytically validate this kit. Data were analyzed using PROC MIXED in SAS 9.4. Plasma cortisol concentrations increased in response to trailering (254.5 ± 26.4 nmol/L, 0 min post-transportation) compared to pre-transportation (142.8 ± 26.4 nmol/L). FCM concentrations increased 24 h post-trailering (10.8 ± 1.7 ng/g) when compared to pre-transportation (7.4 ± 1.7 ng/g). These data support that changes in FCMs can be observed 24 h post-stressor. In conclusion, the Arbor Assays^TM^ DetectX^®^ Cortisol ELISA kit is a reliable, economic option for the measurement of biologically relevant changes in cortisol in equine plasma and FCMs.

## 1. Introduction

Cortisol is commonly measured as a physiological stress indicator. Measuring cortisol does not directly quantify stress. However, as glucocorticoids (GCs) are key mediators of the systemic stress response, cortisol measurements are commonly included as a stress marker [1]. Cortisol is the main GC in mammals [2,3]. Cortisol sampling can be performed with a variety of sample types, including plasma and feces, and there are a variety of pros and cons to each sampling method. The method should reflect the objectives to be assessed.

Blood, plasma, or serum has historically been the medium of choice for GC concentrations as it gives a “snapshot” of the animal at the time of sampling [4,5]. Due to the biological rhythms of this hormone, it is important to control for the time of day that the sample is taken [6,7]. When an animal is exposed to a short-term stressor, like trailering, there is an almost immediate rise in plasma cortisol [8,9]. This makes plasma cortisol an excellent measure of acute stress responses [10]. However, the activity of collecting blood samples may induce a stress response [3,11]. Blood cortisol may not represent an accurate measure for chronic stress as the altered GC production pattern due to sampling stress and time of day must be considered [7,11].

The measurement of fecal cortisol/corticosterone metabolites (FCMs) is a method for assessing the stress response [12,13]. This sampling method is relatively non-invasive, especially when compared to sampling blood. Fecal collection reduces concern for a rise in cortisol from the sampling method as opposed to the treatment. This method has been shown to be a more consistent measure of cortisol in horses without the daily diurnal rhythm observed in blood cortisol that may be useful in assessing animal well-being compared to a particular time point, as the circulating FCMs are incorporated over time and represent an aggregate secretion of hormone [12,13,14]. When considering the collection of samples, the objectives of the study and sample type are important factors to consider. The time it takes for an adrenocortical response to occur, which includes increased GC concentrations in the plasma and corresponding changes in excreted FCMs, varies by species [14]. FCMs are considered a biologically important measure of total GC release, accounting for both intensity and duration of the adrenocortical response [4,15].

However, there are some shortcomings in the ability to process samples, including cost of analysis and sampling timeline. In horses, liquid chromatography with mass spectrometry (LC-MS) has been found to allow for the greatest accuracy compared to immunoassay; however, analytical costs are higher with this method. When comparing differences within animals, immunoassays were found to be acceptable in horses, as long as absolute values/ranges were avoided due to cross-reactivities and potential bias [16]. Commercial enzyme-linked immunoassay (ELISA) kits are a relatively cost-effective and simple method of analysis without having to dispose of radioactive components [17,18]. Thus, ELISA kits lend a potential solution to the cost and ease of analysis. However, the cortisol ELISA kits on the market have not been properly validated for use with equine fecal and/or plasma samples [1]. A review on the non-invasive measurement of glucocorticoids was conducted recently that gives an excellent overview of the advantages and limitations of utilizing feces as a sample matrix [1].

Many commercial ELISA kits have passed analytical validation but lack any physiological/biological validation to show that they can detect a biological change in the sample matrix used (e.g., feces) and within the sample in the species being measured [1]. A biological validation step is crucial to demonstrate that the extraction method and commercial kit used can detect biologically significant changes in adrenocortical activity [1]. It is also important to note that once a particular kit and extraction process is validated for use in a particular species, it does not result in validation for (1) the use of that sample regardless of commercial kit (e.g., feces) and (2) regardless of extraction method for that species [1]. To perform biological validation of commercial ELISA kits, the animal is exposed to a known stressor that will elevate hypothalamic–pituitary–adrenal (HPA) activity for minutes to hours with a rise in a known stress measurement (e.g., plasma cortisol, FCM, or heart rate) [1,13]. Trailering has been shown to induce stress in horses, demonstrated by elevated heart rates and blood and salivary cortisol concentrations determined via ELISA [19,20,21]. Thus, trailering horses is expected to increase blood cortisol concentrations. This is needed to validate the subsequent rise in FCM concentrations, which is expected to occur approximately 24 h after a rise in blood cortisol concentrations [22,23].

The objective of this study was to provide both analytical and biological validation for the detection of biologically relevant changes in FCMs and plasma cortisol from horses using a cost-effective, commercial cortisol ELISA kit (Arbor Assays^TM^ DetectX^®^ Cortisol ELISA kit, K003-H1) that has been validated in other species [24,25,26].

## 2. Materials and Methods

### 2.1. Animals and Husbandry

Quarter horses (*n* = 8) aged 1 to 15 years (mean ± SD: 4 ± 5 yr) consisting of four geldings and four mares were used in this study. The number of horses used was based on using a power test [27]. Horses were a part of the university herd and housed outdoors in paddocks at the Ohio State University Equine Facility, Columbus campus in Columbus, Ohio. All horses were healthy and sound prior to and during the study. The study was conducted in October of 2022 and the horses were not ridden or in training during the study. Horses were fed forage and a commercial concentrate to maintain body condition. Water and mineral blocks were provided ad libitum. The study was approved by the Ohio State University Institutional Animal Care and Use Committee (IACUC: 2022A00000045).

Horses were transported untied, in pairs, for a 15 min transportation session using a livestock trailer that included a divider to prevent physical contact and limit visual contact. Two trips took place on two consecutive days to ensure that the horses were transported during the same time of day. Both 15 min transports were completed within a 30 min period of time to reduce the chances of differences in plasma cortisol levels due to circadian rhythm. Each horse was randomly assigned to a day and transported once during the study. The driver, truck, and trailer were the same for every trip and the same route was used. All horses had been transported prior to the study, but not consistently or for long periods of time. Horses were brought into stalls prior to trailering to obtain baseline fecal samples.

### 2.2. Blood Collection and Analysis

**Figure 1:**
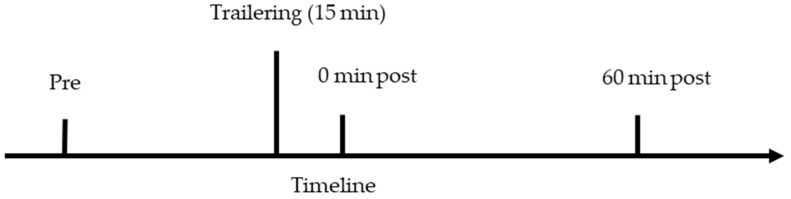
Blood sampling timeline relative to transportation of horses for 15 min in a trailer. Blood samples were obtained prior to transportation (pre), immediately after transportation (0 min post), and 60 min after transportation (60 min post).

Blood samples were collected via jugular venipuncture before (baseline), immediately after, and 60 min post-transportation (Figure 1). The baseline blood sample was taken in the area where the horses were housed, prior to the horses coming to be transported to avoid any possible stress of temporary stalling that would result in a rise of plasma cortisol. Blood samples were collected into a tube (BD Vacutainer™, Franklin Lakes, NJ, USA) containing sodium heparin to allow for separation of plasma and to reduce the chance of interaction with the assay used [28]. After inverting several times, blood samples were stored at 4 °C for no more than 1 h prior to plasma extraction. Plasma was obtained by centrifuging the blood samples at 1500× *g* for 10 min at 4 °C. Two aliquots of 1 to 1.5 mL were frozen at −20 °C until analysis [29]. This protocol was determined based on previous literature and the manufacturer’s recommendations [30].

For hormone analysis, 5 µL of Dissociation Reagent (Arbor Assays, Ann Arbor, MI, USA) was added to a clean 1.5 mL tube according to the manufacturer’s instructions. A 5 µL plasma sample was also added to the tube, vortexed, and incubated at room temperature for at least 5 min. Assay buffer (Arbor Assays^TM^, Ann Arbor, MI, USA) was then added, and plasma cortisol concentrations (PCC, ng/mL) were determined using a commercial cortisol enzyme immunoassay kit (Arbor Assays^TM^ DetectX^®^ Cortisol ELISA kit, K003-H1, Ann Arbor, MI, USA) and ran in duplicate.

### 2.3. Fecal Collection and Analysis

**Figure 2:**
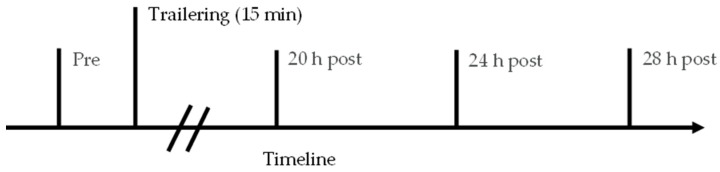
Fecal sampling timeline relative to transportation of horses for 15 min in a trailer. Fecal samples were obtained prior to transportation (pre), 20 h after transportation (20 h post), 24 h after transportation (24 h post), and 28 h after transportation (28 h post).

Fecal samples were collected via drop or grab sampling prior to transportation and 20, 24, and 28 h post-transportation (Figure 2). Due to the timeline for measuring FCMs, if fecal samples were not collected from drop samples within 15 min of the stated sampling time, a sample was taken directly from the rectum using a sleeve over the hand and arm of the sampler. Only one fecal sample was taken directly from the rectum. A minimum of 100 g of feces was collected at each time point. Samples were thoroughly homogenized to ensure that FCMs were distributed equally throughout the feces [22]. After homogenization, fecal samples were split into 2 aliquots of at least 50 g, and frozen at −20 °C within 10 min of defecation. Fecal samples were stored at −20 °C until hormone extraction and analysis to avoid further bacterial metabolism of FCMs [17,31,32]. Frozen fecal samples were put into an oven at 55 °C for 48 h without thawing first to prevent both microbial metabolism and heat damage [12,17,33,34,35]. At 24 h, the fecal samples were manually broken up to help ensure even, complete drying of the feces. Dried fecal samples were ground using a coffee grinder and split into four aliquots (0.20 g each). Hormones were extracted from the fecal samples in glass tubes with Teflon caps. An amount of 1.0 mL of 90% ethanol per 0.1 g solid (2 mL for 0.2g feces) was added [14,19,28,33]. Samples were shaken vigorously at 37 °C for 30 min using a commercial incubator shaker and then centrifuged at 3300× *g* for 15 min at 4 °C. The supernatant was decanted into clean 1.5 mL tubes and centrifuged again at 3300× *g* for 15 min at 4 °C to ensure no solids remained. An amount of 600 µL of supernatant was collected into a clean 1.5 to 2.0 mL tube and evaporated under nitrogen to dryness to avoid steroid hormone oxidation [36,37]. Extracts were stored at −80 °C until hormone analysis. This procedure was created specifically for the extraction of equine FCMs with consideration given to previous literature [24,25,26].

Extracted samples were dissolved into 100 µL of 90% ethanol, vortexed, and rested for 5 min. This was repeated two additional times to ensure steroid solubility. Assay buffer (Arbor Assays^TM^, Ann Arbor, MI, USA) was then added, and FCM concentrations (ng/g feces) were determined using a commercial cortisol enzyme immunoassay kit (Arbor Assays^TM^ DetectX^®^ Cortisol ELISA kit, K003-H1, Ann Arbor, MI, USA) and ran in duplicate. Due to the uncertainty of how much native molecules and reactive metabolites were quantified using this kit, the term FCM concentration will be used.

### 2.4. Data Analysis

Analytical validation was evaluated for precision (inter-assay and intra-assay coefficients of variation, % CV), parallelism, and accuracy (percent recovery of a known spike) [14]. Precision (% CV of intra- and inter-assay of the standard control) was calculated using the formula [(standard deviation/average) × 100]. Parallelism was determined by comparing the equality of slope between the sample curve and the standard curve to demonstrate a dose–response relationship. Accuracy (% recovery) was calculated using the formula [(measured concentration/known concentration) × 100]. According to the manufacturer, cross-reactivity is as follows: cortisol 100%, dexamethasone 18.8%, prednisolone (1-dehydrocortisol) 7.8%, corticosterone 1.2%, cortisone 1.2%, progesterone < 0.1%, estradiol < 0.1%, cortisol 21-glucuronide < 0.1%, 1α-hydroxycorticosterone < 0.1%, and testosterone < 0.1%. However, cross-reactivity on various FCMs has not been described.

Based on manufacturer’s recommendation, the suggested dilution of plasma was 1:100 with reagent diluent provided by the manufacturer. A recommended dilution for feces was not provided, only that the final ethanol concentration would be less than 5%. Therefore, serial dilutions of 1:100, 1:150, 1:200, and 1:250 of plasma samples and 1:10, 1:20, 1:40, and 1:80 of fecal samples were used to test for dilutional parallelism. Four random samples each were chosen to perform serial dilutions for both sample matrices. The manufacturer’s recommended standard curve was developed using the provided cortisol stock solution (Arbor Assays, Ann Arbor, MI, USA) at concentrations of 11.10, 5.55, 2.77, 1.39, 0.69, 0.35, 0.17, and 0 nmol/L.

Plasma from three random samples was diluted 1:200 with either reagent diluent or reagent diluent spiked with a known amount of cortisol standard (1.11, 2.22, and 4.44 nmol/L; Arbor Assays, Ann Arbor, MI, USA). Four random fecal samples were diluted 1:40 with either reagent diluent or reagent diluent spiked with a known amount of cortisol standard (0.55, 1.11, and 2.22 nmol/L; Arbor Assays, Ann Arbor, MI, USA). Percent recovery was determined by comparing observed and expected concentrations (Table 1).

Biological validation was performed by comparing plasma cortisol concentrations (PCCs) and FCM concentrations before and after transport. For these data, the horse was the experimental unit and was treated as a random variable nested within trip nested within sex, along with trip nested within sex. Data were analyzed using PROC MIXED with a repeated measures statement in SAS 9.4 (SAS Institute Inc., Cary, NC, USA) with time, sex, and their interaction (Sex × Time) as fixed effects. The covariance matrix Toeplitz structure (2) was chosen as it produced the lowest Akaike information criterion corrected (AICC). For this study, data were considered significant at *p* ≤ 0.05 and a significant trend is described when *p* > 0.05 and ≤0.10.

## 3. Results

### 3.1. Validation Tests

The average recoveries of a known spike of cortisol in plasma and FCMs were 98.2 and 100.0%, respectively (Table 1). Average inter-assay and intra-assay coefficients of variation for plasma cortisol were 11.0 and 7.2%, respectively. Average inter-assay and intra-assay coefficients of variation for FCMs were 9.0 and 10.8%, respectively.

**Figure 3:**
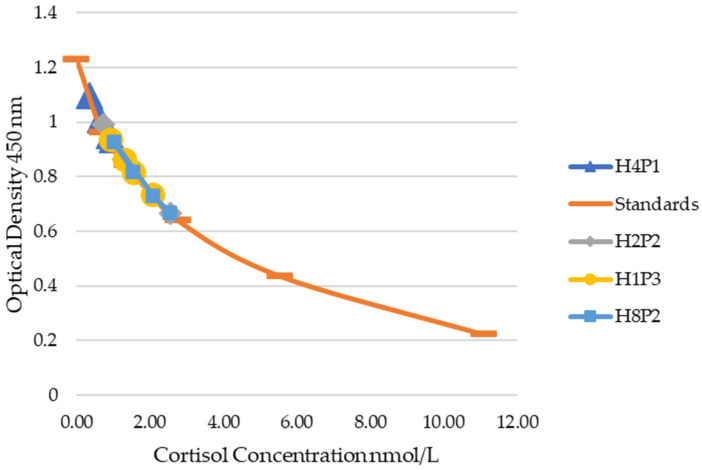
Dilutional parallelism of four individual plasma samples against the standards. From left, 1:250, 1:200, 1:150, and 1:100 dilutions.

**Figure 4:**
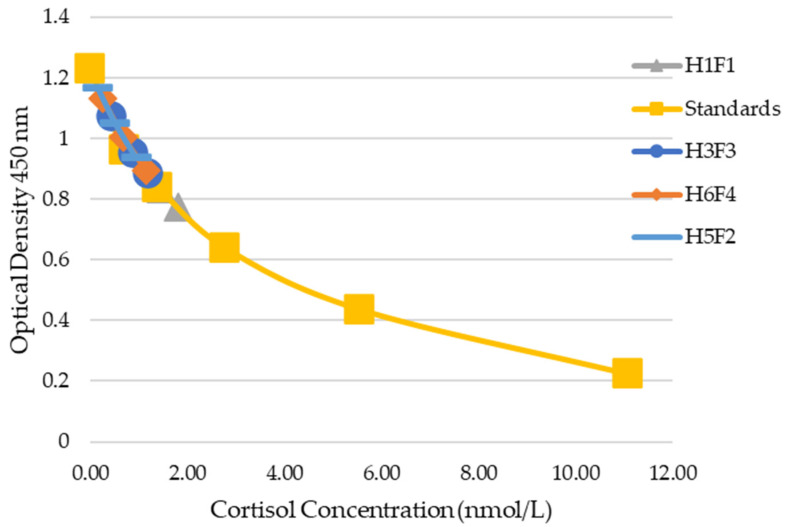
Dilutional parallelism of four individual fecal samples against the standards. From left, 1:80, 1:40, and 1:20 dilutions.

For plasma samples, dilutional parallelism was observed for 1:100, 1:150, 1:200, and 1:250 (Figure 3). Dilutional parallelism was observed between dilutions 1:20, 1:40, and 1:80 for fecal samples (Figure 4). Dilutions of 1:40 for fecal samples and 1:200 for plasma samples were chosen for further evaluation. The limit of detection was determined to be 0.35 nmol/L as there were no observed differences between 0, 0.17, and 0.35 nmol/L standard values. 

### 3.2. Plasma Cortisol Concentrations

**Figure 5:**
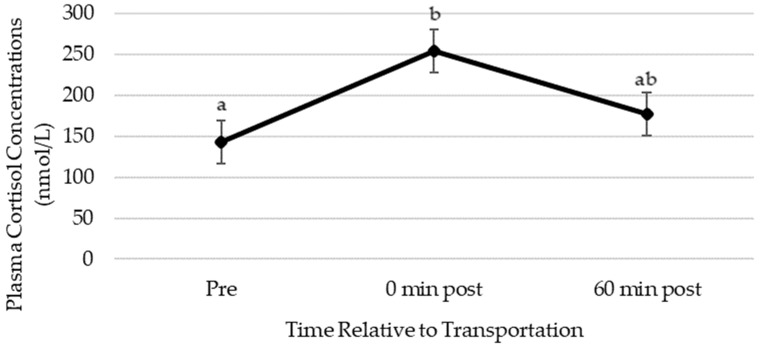
Least squares mean (±SEM) for plasma cortisol concentrations in equine plasma pre- and post-transportation. Different letters indicate significant differences (a, b; *p* ≤ 0.05). SEM, standard error of the mean.

Plasma cortisol concentrations increased in response to trailering (254.5 ± 26.4 nmol/L, 0 min post-transportation) compared to pre-transportation (142.8 ± 26.4 nmol/L) and showed a tendency to decline 60 min post-transportation (176.8 ± 26.4 nmol/L) when compared to immediately post-trailering (Figure 5). Plasma cortisol concentrations pre-trailering were similar to 60 min post-trailering. There were no differences in plasma cortisol concentrations due to trip or sex.

### 3.3. FCM Concentrations

**Figure 6:**
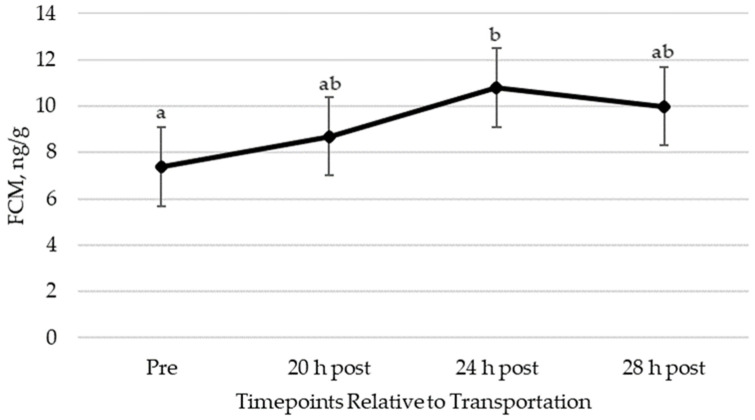
Least squares mean (±SEM) for fecal cortisol/corticosterone metabolite (FCM) concentrations in equine feces pre- and post-transportation. Different letters indicate significant differences (a, b; *p* ≤ 0.05). SEM, standard error of the mean.

FCM concentrations changed in response to transportation (Figure 6). FCM concentrations increased 24 h post-trailering (10.8 ± 1.7 ng/g) when compared to pre-transportation (7.4 ± 1.7 ng/g). FCM concentrations pre-trailering were not different from 20 h (8.7 ± 1.7 ng/g) or 28 h post-trailering (10.0 ± 1.7 ng/g). There were no differences in FCM concentrations due to trip or sex.

## 4. Discussion

Cortisol has long been used to evaluate animal stress, and in a larger sense, animal welfare [1,7,38]. ELISA kits have grown in popularity as a way to measure cortisol in various samples, such as plasma, feces, and saliva, and the need to validate these kits has also increased. Analytical validations comprise measures of precision, sensitivity, specificity, and accuracy and are crucial components to allow for accurate interpretation of results. Spike and recovery test results indicate that other components of the sample matrices utilized did not interfere with the estimation of cortisol concentrations, while % CV indicates an acceptable level of precision. The results from the test of dilutional parallelism demonstrated that both sample matrices for this ELISA interact with the assay antibody in a dose-dependent manner and thus support that the standards and samples have similar antibody binding characteristics. However, it is possible for a kit to be analytically validated without the ability to detect a biological signal in the sample matrices [1].

To biologically validate an assay measuring cortisol, a demonstrated increase in cortisol is required in response to a stressor. These data support previous research whereby providing transportation via horse trailer for 15 min with limited visual and physical contact with other horses was sufficient to produce a stress response observed in plasma [20,21,30]. Previous research showed that transportation varying from 1 to 8 h resulted in a stress response measured via fecal samples [19,30]. However, this study showed that a shorter timeframe (15 min) with limited visual/physical contact between conspecifics was adequate to elicit an acute stress response in horses and is appropriate to use in biological validation methods that require a stress response reflected in both plasma and feces.

Transporting horses is a common practice as owners engage in equine recreational activities, including various competitions, trail rides, and more [39]. Transport is generally less than 8 h and various studies have found that transport is considered stressful by the horse using a variety of techniques (e.g., plasma cortisol, FCM, salivary cortisol, and heart rate variability [HRV]) [8,40,41,42]. Notably, Schmidt and colleagues [8] found that the degree of changes to cortisol release and HRV were proportionate to the length of transport. Longer transports were more stressful than shorter ones [8]. In a similar study, it was observed that transport-naïve horses experienced a greater level of stress that decreased with exposure to transport [43]. Some research indicates as little as 15 min of transport is sufficient to result in a significant rise in blood cortisol in horses [21].

In this study, increases in plasma cortisol were observed at the time of the stressor and in FCM concentrations 24 h post-stressor. These data support that changes in plasma cortisol in response to a stressor are observed immediately after the stressor [44]. These data also support that changes in FCMs occur approximately 24 h after a stress-inducing challenge (e.g., trailering) [2,13,22]. Thus, it may be important to collect several samples over time to ensure that an environmental stressor (single event) does not skew the overall results if investigating long-term stress. Equine FCM concentrations have ranged between 1.3 and 121.4 ng/g, which is a large range, possibly due to differences in sampling methods, metabolite measured, method of analysis, environment, diet, storage methods, and stressors [11,31,45,46,47,48,49,50]. Due to the lack of data on cross-reactivity to FCMs for this commercial kit, the term FCM was chosen to better describe what this commercial cortisol ELISA kit may detect. The values for FCM concentrations were similar to some previous literature in horses [11,48,51] but were lower than others [8]. This may be due to a difference in cross-reactivity of certain fecal cortisol metabolites across different analyses or different variations of stressors used (e.g., longer transportation periods). Further research is needed to determine the extent of cross-reactivity with various FCMs in this commercial kit and what these concentrations represent.

## 5. Conclusions

In conclusion, the commercial ELISA kit evaluated in this study can be used as a reliable, economic option for the measurement of a biologically relevant change in equine plasma cortisol and FCMs. In the present study, increases in plasma cortisol were observed at the time of the stressor and in FCM concentrations approximately 24 h post-stressor. Further research should be conducted to examine the cross-reactivity of equine FCMs and this commercial kit.

## Figures and Tables

**Table 1:** Average concentrations (range) of cortisol in equine plasma and feces diluted with reagent spiked with cortisol.

Plasma				Feces			
Spike, nmol/L	Observed, nmol/L	Expected, nmol/L	Recovery, %	Spike, nmol/L	Observed, nmol/L	Expected, nmol/L	Recovery, %
0	0.98 (0.24–1.84)	0.98 (0.24–1.84)	100.0 (100.0)	0	1.01 (0.35–1.83)	1.01 (0.35–1.83)	100.0 (100.0)
1.11	2.42 (1.96–3.10)	2.09 (1.35–2.95)	99.7 (99.4–99.9)	0.55	1.37 (0.81–1.90)	1.56 (0.91–2.38)	100.2 (99.8–100.5)
2.22	3.07 (2.56–3.52)	3.20 (2.46–4.06)	100.1 (99.5–100.5)	1.11	2.46 (1.84–3.34)	2.12 (1.46–2.93)	99.7 (99.1–100.1)
4.44	5.74 (4.64–6.42)	5.42 (4.68–6.28)	99.7 (99.2–100.1)	2.22	3.05 (2.32–3.44)	3.23 (2.57–4.05)	100.2 (99.5–100.6)

## Data Availability

The data presented in this study can be requested from the corresponding author. The data are not publicly available.

## References

[1] Palme R. (2019). Non-invasive measurement of glucocorticoids: Advances and problems. Phys. Behav..

[2] Merl S., Scherzer S., Palme R., Möstl E. (2020). Pain causes increased concentrations of glucocorticoid metabolites in horse feces. J. Equine Vet. Sci..

[3] Mormède P., Andanson S., Aupérin B., Beerda B., Guémené D., Malmkvist J., Manteca X., Manteuffel G., Prunet P., van Reenen C.G. (2007). Exploration of the hypothalamic–pituitary–adrenal function as a tool to evaluate animal welfare. Phys. Behav..

[4] Sheriff M.J., Dantzer B., Delehanty B., Palme R., Boonstra R. (2011). Measuring stress in wildlife: Techniques for quantifying glucocorticoids. Oecologia.

[5] Spencer R.L., Deak T. (2017). A users guide to HPA axis research. Phys. Behav..

[6] Van Cauter E., Leproult R., Kupfer D.J. (1996). Effects of gender and age on the levels and circadian rhythmicity of plasma cortisol. J. Clin. Endocrinol. Metab..

[7] Palme R. (2012). Monitoring stress hormone metabolites as a useful, non-invasive tool for welfare assessment in farm animals. Anim. Welf.

[8] Schmidt A., Möstl E., Wehnert C., Aurich J., Müller J., Aurich C. (2009). Cortisol release and heart rate variability in horses during road transport. Horm. Behav..

[9] Bennett-Wimbush K., Suagee-Bedore J., Amstutz M. (2018). PSXVI-17 Acclimation Reduces Stress in Adult Horses Tied in Visual Isolation. J. Anim. Sci..

[10] Sikorska U., Maśko M., Ciesielska A., Zdrojkowski Ł., Domino M. (2023). Role of Cortisol in Horse’s Welfare and Health. Agriculture.

[11] Fureix C., Benhajali H., Henry S., Bruchet A., Prunier A., Ezzaouia M., Coste C., Hausberger M., Palme R., Jego P. (2013). Plasma cortisol and faecal cortisol metabolites concentrations in stereotypic and non-stereotypic horses: Do stereotypic horses cope better with poor environmental conditions?. BMC Vet. Res..

[12] Möstl E., Palme R. (2002). Hormones as indicators of stress. Dom. Anim. Endocrin..

[13] Touma C., Palme R. (2005). Measuring fecal glucocorticoid metabolites in mammals and birds: The importance of validation. Ann. N. Y. Acad. Sci.

[14] Palme R. (2005). Measuring fecal steroids: Guidelines for practical application. Ann. N. Y. Acad. Sci..

[15] Keay J.M., Singh J., Gaunt M.C., Kaur T. (2006). Fecal glucocorticoids and their metabolites as indicators of stress in various mammalian species: A literature review. J. Zoo Wildl. Med..

[16] Saluti G., Ricci M., Castellani F., Colagrande M.N., Di Bari G., Vulpiani M.P. (2022). Determination of hair cortisol in horses: Comparison of immunoassay vs LC-HRMS/MS. Anal. Bioanal. Chem..

[17] Möstl E., Rettenbacher R., Palme R. (2005). Measurement of corticosterone metabolites in birds’ droppings: An analytical approach. Ann N.Y. Acad Sci.

[18] Kersey D.C., Dehnhard M. (2014). The use of noninvasive and minimally invasive methods in endocrinology for threatened mammalian species conservation. Gen. Comp. Endocrinol..

[19] Schmidt A., Hödl S., Möstl E., Aurich J., Müller J., Aurich C. (2010). Cortisol release, heart rate, and heart rate variability in transport-naive horses during repeated road transport. Dom. Anim. Endocrin..

[20] Padalino B. (2015). Effects of the different transport phases on equine health status, behavior, and welfare: A review. J. Vet. Behav..

[21] Heitman K., Rabquer B., Heitman E., Streu C., Anderson P. (2018). The use of lavender aromatherapy to relieve stress in trailered horses. J. Eq. Vet. Sci..

[22] Palme R., Fischer P., Schildorfer H., Ismail M.N. (1996). Excretion of infused 14C-steroid hormones via faeces and urine in domestic livestock. Anim. Repro. Sci..

[23] Möstl E., Messmann S., Bagu E., Robia C., Palme R. (1999). Measurement of glucocorticoid metabolite concentrations in faeces of domestic livestock. J. Vet. Med..

[24] Cornale P., Macchi E., Miretti S., Renna M., Lussiana C., Perona G., Mimosi A. (2015). Effects of stocking density and environmental enrichment on behavior and fecal corticosteroid levels of pigs under commercial farm conditions. J. Vet. Behav..

[25] Pecorella I., Ferretti F., Sforzi A., Macchi E. (2016). Effects of culling on vigilance behaviour and endogenous stress response of female fallow deer. Wildl. Res..

[26] Gholib G., Wahyuni S., Wahyudi A., Silalahi K.S., Akmal M., Sabri M., Nugraha T.P. Validation of commercial ELISA kit for non-invasive measurement of cortisol concentrations and the evaluation of the sampling time of blood and fecal sample in Aceh cattle. Proceedings of the 1st International Conference on Veterinary, Animal, and Environmental Sciences (ICVAES 2019).

[27] Arifin W.N., Zahiruddin W.M. (2017). Sample Size Calculation in Animal Studies Using Resource Equation Approach. Malays. J. Med. Sci. MJMS.

[28] Nielsen E.W., Johansen H.T., Straume B., Mollnes T.E. (1994). Effect of time, temperature and additives on a functional assay of C1 inhibitor. J. Immunol. Methods.

[29] Stroud L.R., Solomon C., Shenassa E., Papandonatos G., Niaura R., Lipsitt L.P., LeWinn K., Buka S.L. (2007). Long-term stability of maternal prenatal steroid hormones from the National Collaborative Perinatal Project: Still valid after all these years. Psychoneuroendocrinology.

[30] Thomson T.L. (2020). The Effect of Citrus Botanical Oil on Equine Behavior. PhD Dissertation.

[31] Morrow C.J., Kolver E.S., Verkerk G.A., Matthews L.R. (2002). Fecal glucocorticoid metabolites as a measure of adrenal activity in dairy cattle. Gen. Comp. Endocrinol..

[32] Yarnell K., Purcell R.S., Walker S.L. (2016). Fecal glucocorticoid analysis: Non-invasive adrenal monitoring in equids. J. Vis. Exp. JoVE.

[33] Gholib G., Heistermann M., Agil M., Supriatna I., Purwantara B., Nugraha T.P., Engelhardt A. (2018). Comparison of fecal preservation and extraction methods for steroid hormone metabolite analysis in wild crested macaques. Primates.

[34] Palme R., Robia C., Baumgartner W., Möstl E. (2000). Transport stress in cattle as reflected by an increase in faecal cortisol metabolite concentrations. Vet. Rec..

[35] Santos J.P., Vicente J., Villamuelas M., Albanell E., Serrano E., Carvalho J., Fonseca C., Gortazar C., López-Olvera J.R. (2014). Near infrared reflectance spectroscopy (NIRS) for predicting glucocorticoid metabolites in lyophilised and oven-dried faeces of red deer. Ecol. Ind..

[36] Brown J.L., Wasser S.K., Wildt D.E., Graham L.H. (1994). Comparative aspects of steroid hormone metabolism and ovarian activity in felids, measured noninvasively in feces. Biol. Reprod..

[37] Sadoul B., Geffroy B. (2019). Measuring cortisol, the major stress hormone in fishes. J. Fish. Biol..

[38] Broom D.M. (2001). Coping, stress and welfare. Coping with Challenge: Welfare in Animals Including Humans, Proceedings of Dahlem Conference, ed. Broom DM, 1-9 Berlin, Germany 2000.

[39] Friend T.H. (2001). A review of recent research on the transportation of horses. J. Anim. Sci..

[40] Stull C.L., Spier S.J., Aldridge B.M., Blanchard M., Stott J.L. (2004). Immunological response to long-term transport stress in mature horses and effects of adaptogenic dietary supplementation as an immunomodulator. Equine Vet. J..

[41] Fazio E., Medica P., Cravana C., Giacoppo E., Ferlazzo A. (2009). Physiological variables of horses after road transport. Animal.

[42] Roy R.C., Cockram M.S., Dohoo I.R. (2015). Welfare of horses transported to slaughter in Canada: Assessment of welfare and journey risk factors affecting welfare. Can. J. Anim. Sci..

[43] Schmidt A., Biau S., Möstl E., Becker-Birck M., Morillon B., Aurich J., Faure J.-M., Aurich C. (2010). Changes in cortisol release and heart rate variability in sport horses during long-distance road transport. Domest. Anim. Endocrinol..

[44] Matteri R.L., Carroll J.A., Dyer C.J. (2000). Neuroendocrine responses to stress. The Biology of Animal Stress: Basic Principles and Implications for Animal Welfare.

[45] Berghold P., Möstl E., Aurich C. (2007). Effects of reproductive status and management on cortisol secretion and fertility of oestrous horse mares. Anim. Reprod. Sci..

[46] Gorgasser I., Tichy A., Palme R. (2007). Faecal cortisol metabolites in Quarter Horses during initial training under field conditions. Wien. Tierarztl. Monatsschrift.

[47] Flauger B., Krueger K., Gerhards H., Möstl E. (2010). Simplified method to measure glucocorticoid metabolites in faeces of horses. Vet. Res. Commun..

[48] Pawluski J., Jego P., Henry S., Bruchet A., Palme R., Coste C., Hausberger M. (2017). Low plasma cortisol and fecal cortisol metabolite measures as indicators of compromised welfare in domestic horses (*Equus caballus*). PLoS ONE.

[49] van Vollenhoven E., Grant C.C., Fletcher L., Schulman M.L., Page P.C., Ganswindt A. (2018). Salivary glucocorticoid and fecal glucocorticoid metabolite concentrations in pony mares during transrectal palpation of the reproductive tract by veterinary students. J. Equine Vet. Sci..

[50] Christensen J.W., Strøm C.G., Nicová K., de Gaillard C.L., Sandøe P., Skovgård H. (2022). Insect-repelling behaviour in horses in relation to insect prevalence and access to shelters. Appl. Anim. Behav. Sci..

[51] Mercer-Bowyer S., Kersey D.C., Bertone J.J. (2017). Use of fecal glucocorticoid and salivary cortisol concentrations as a measure of well-being of New York City carriage horses. J. Am. Vet. Med. Assoc..

